# Lipase-Catalyzed Interesterification for the Synthesis of Medium-Long-Medium (MLM) Structured Lipids – A Review

**DOI:** 10.17113/ftb.57.03.19.6025

**Published:** 2019-09

**Authors:** Qabul Dinanta Utama, Azis Boing Sitanggang, Dede Robiatul Adawiyah, Purwiyatno Hariyadi

**Affiliations:** 1 Department of Food Science and Technology, Faculty of Agricultural Engineering and Technology, IPB University (Bogor Agricultural University), Darmaga, 16680 Bogor, Indonesia; 2 Southeast Asian Food and Agricultural Science and Technology (SEAFAST) Center, IPB University (Bogor Agricultural University), Darmaga, 16680 Bogor, Indonesia

**Keywords:** glycerides, interesterification, lipase, medium-long-medium structured lipids

## Abstract

Medium-long-medium (MLM) structured lipids typically contain medium-chain fatty acids (C6-C12) at *sn*-1,3 and long-chain fatty acids (C14-C24) at *sn*-2 positions. They have reduced calories and are suitable for the control of obesity, lipid malabsorption and other metabolic disorders. This review focuses on the synthesis of MLM lipids by the enzymatic interesterification. It gives detailed description of biocatalysts, substrates, reactors and synthesis methods, and discusses the use of MLM lipids in food products. The information provided in this review can be considered as the current state-of-the art for developing a future strategy for the synthesis of MLM structured lipids.

## INTRODUCTION

The carbon chain length, number of double bonds, and the position of fatty acids in triacylglycerol (TAG) molecules are very important factors affecting physicochemical, functional and nutritional properties of lipids (*1*, *2*). Long-chain fatty acids (LCFA: C14-C24), especially monounsaturated fatty acids (MUFA) and polyunsaturated fatty acids (PUFA), have been shown to have health benefits for human body (*3*). PUFA are widely known as essential fatty acids (*4*). The presence of PUFA in human body plays important roles for the prevention of various diseases and disorders, such as cardiovascular disease, inflammation, allergy, cancer, immune response, diabetes, hypertension and renal disorders (*3*). Furthermore, medium-chain fatty acids (MCFA: C6-C12) have been identified as quick energy sources as they are easily transported to the liver for the production of ketones (*5*–*7*). Vandenberghe *et al.* (*8*) reported that both caprylic (C8:0) and capric acid (C10:0) are effective in increasing plasma ketones, and may potentially provide constant energy for the body. In addition, it has been reported that MCFA also have a weak tendency to accumulate in adipose tissue (*6*, *9*–*11*).

The position of MCFA and LCFA in a TAG affects the digestion and subsequent absorption of the fatty acids. An MCFA that is located at *sn-*1,3 position is easily hydrolyzed by the lipase, shown by a higher possibility to be absorbed than any LCFA (*5*, *12*, *13*). On the other hand, LCFAs located at *sn*-1,3 position are more likely to react with calcium to form soap (*1*). Stuctured lipids containing combinations of LCFA at *sn*-2 and MCFAs at *sn*-1,3 position, so-called medium-long-medium (MLM) TAG, will provide lipids with high coeficient absorption (*14*, *15*). It has been reported that MLM structured lipids have a low caloric value, and can be used to control obesity, fat malabsorption, and other metabolic disorders (*2*, *16*–*18*). However, an MLM lipid is rarely found in nature in a high concentration, therefore, there is a need for developing its preparation.

MLM structured lipids as reported in the literature are mostly synthesized by enzymatic interesterification, as compared to chemical interesterification. The enzymatic interesterification has some advantages due to its specificity and mild reaction conditions, hence producing fewer byproducts (*19*–*22*). Hereby, this review will focus on the underlining factors for the enzymatic interesterification for production of MLM TAGs, including the biocatalysts, substrates, reactor configurations, synthesis methods, and other reaction conditions. Additionally, this review also presents the potential applications of MLM structured lipids, particularly in food industry.

## ENZYMES FOR MLM STRUCTURED LIPID SYNTHESIS: TYPES ANS SOURCES

Lipases are enzymes belonging to the hydrolase group, capable of catalyzing both hydrolysis and esterification reactions (*23*–*25*). A lower moisture content of reaction system provides a greater possibility for lipases to catalyze esterification reaction than that of hydrolysis reaction. However, the presence of small quantities of moisture is still needed for the lipases to maintain their catalytic activities (*26*). Generally, the active site of a lipase is responsible for its catalytic activity and it consists of a triad of serine, histidine and aspartate or glutamate (His-Ser-Asp/Glu). This catalytic triad is buried under the ‘lid’ of the surface circle that undergoes conformational changes due to interfacial activation. These conformational changes result in the availability of open channels for facilitating active sites that are correctly oriented for the substrates (*27*).

The mechanism of lipase-catalyzed esterification consists of three steps (*24*). In the first step, the active sites of serine are activated by deprotonation using histidine and aspartate. Further, the serine active sites react with the carbonyl group of the substrates forming an acyl-enzyme intermediate, stablized by oxyanion hole. Finally, such deacylation is performed where a nucleophile (*e.g*. H_2_O or monoglyceride) attacks acyl-enzyme intermediate to release a product and regenerate unoccupied catalytic sites. Electronegativity of the molecules populating the interface controls this process (*24*).

Animals, plants and microbes (fungi, yeast and bacteria) have been identified as sources of lipases (*28*). Generally, microbial lipases have higher stability than plant and animal lipases (*29*). There are numerous studies of the characteristics of microbial lipases (*i.e*. mostly extracellular bacterial and fungal lipases), and their versatility makes them very attractive for industrial applications (*30*).

**Fig. 1:**
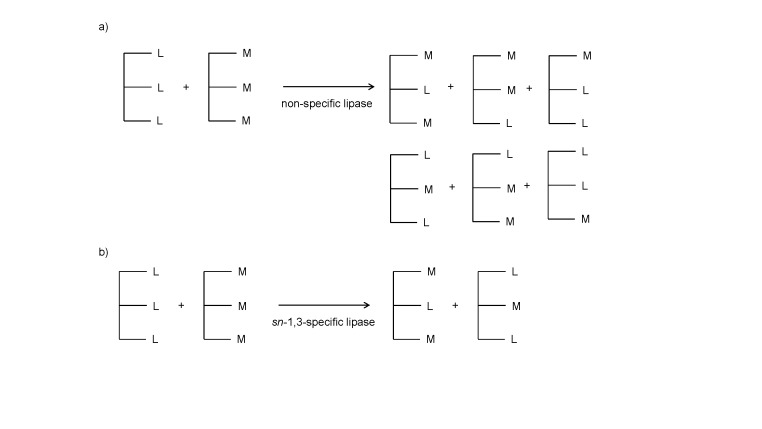
Synthesis of medium-long-medium (MLM) structured lipids by: a) non-specific lipase, and b) *sn*-1,3-specific lipase

In general, based on their mode of action, lipases can be classified as non-specific and specific lipases. A non-specific lipase acts randomly and produces a similar mixture of products to that of a chemical interesterification (*16*). On the other hand, a specific lipase acts uniquely towards producing specific type of product, and can be subclassified based on positional, substrate or stereo specificity (*31*, *32*). An *sn-*1,3-specific lipase, a lipase that has preference to react solely with acyl groups at *sn*-1,3 positions (Fig. 1), is widely used as a catalyst for MLM lipase synthesis. Lipases from *Thermomyces lanuginosa* and *Rhizomucor miehei* have been identified to have *sn-*1,3 positional specificity (*19*). Additionally, several studies have reported that lipases from *Geotrichum candidum* (*33*) and *Candida antartica* (*34*, *35*) show a moderate preference for *sn*-2 position. A stereospecific lipase hydrolyses fatty acids at *sn*-1 and *sn*-3 positions at different rates. Therefore, besides the influence of its source, the catalytic activity largely depends on the substrate concentration (*31*, *32*). On the other hand, a substrate-specific lipase shows specificity to certain fatty acids (*i.e*. saturated or unsaturated fatty acids, long, medium or short chain fatty acids) and the type of acylglycerols (mono-, di-, or tri-acylglycerol). Rodrigues and Fernandez-Lafuente (*36*) reported that *Rhizomucor miehei* lipase showed a high activity in acidolysis, while *Thermomyces lanuginosa* lipase in alcoholysis or transesterification reaction. The specificity of either positional, substrate or stereospecific lipase is also affected by the solvent polarity or partition coefficient (log *P*) (hydrophobic/hydrophilic coefficient), water activity (*a*_w_), immobilization carrier, and reaction conditions (*37*, *38*).

The stability of enzymes is an important aspect for industrial applications due to their high cost. Lipase stability is affected by the reaction conditions, including moisture content (*a*_w_), pH, temperature, substrate composition, product concentration and lipase concentration (*25*, *32*). The enzyme immobilization and enzyme modifications through protein/genetic engineering are an interesting area to increase enzyme stability. Immobilized lipases show higher stability at elevated temperatures in microaqueous systems than in free form (*26*). Several commercial immobilized lipases, including Lipozyme RM IM (from *Rhizomucor miehei*) (*39*-*41*), Lipozyme TL IM (from *Thermomyces lanuginosa*) (*38*, *42*, *43*), and Novozyme 435 (*44*), have been widely used for investigations. Lipozyme TL IM has a lower price than other commercial lipases (*22*). Furthermore, its use in batch reaction effectively changes the TAG composition of palm olein, yet shows high acyl migration (*45*). Cao *et al.* (*46*) also confirmed that this lipase, as compared to that from *Rhizopus oryzae*, has a tendency to cause acyl migration. Lipozyme TL IM has a higher total transesterification activity than Lipozyme RM IM (*39*). Moreover, *sn*-1,3 positional specificity of Lipozyme RM IM is slightly higher than that of Lipozyme TL IM (*42*). Nunes *et al.* (*47*) reported that the highest incorporation of caprylic acid into olive oil was obtained by using Novozyme 435 and Lipozyme RM IM as catalysts. Khodadadi *et al.* (*43*) also reported that Lipozyme TL IM and Novozyme 435 were more effective in transesterification reaction between flaxseed oil and tricaprylin than Lipozyme RM IM and Amano DF. Table 1 (*44*, *48*-*53*) summarizes the utilization of immobilized lipase for MLM lipase synthesis.

**Table 1:** Immobilized lipase for the synthesis of medium-long-medium (MLM) structured lipid

Enzyme	Source	Specific/non-specific	Immobilization material	Reference
Lipase Lip2	*Yarrowia lipolityca*	*sn*-1,3 specific	Accrurel MP 1000	(*44*)
Lipozyme TL IM	*Thermomyces lanuginosa*	*sn*-1,3 specific	Silica gel	(*48*)
Lipozyme RM IM	*Rhizomucor miehei*	*sn*-1,3 specific	Resin	(*49*)
Novozyme 435	*Candida antartica*	*sn*-1,3 specific	Acrylic resin	(*49*, *50*)
Lipozyme 435	Recombinant lipase from *Candida* antartica, expressed on *Aspergillus niger*	*sn*-1,3 specific	Macroporous hydrophobic resin	(*49*)
Lipase D and DF	*Rhizopus oryzae*	*sn*-1,3 specific	Accrurel MP 1000	(*51*)
Lipase QLM	*Alcaligenes* sp.	*sn*-1,3 specific	Accrurel MP 1000	(*51*)
Palatase 20000L	*Mucor miehei*	*sn*-1,3 specific	Accrurel MP 1000	(*52*)
Lipase Rd	*Rhizopus delemar*	*sn*-1,3 specific	Accrurel MP 1000	(*52*)
*Carica papaya* lipase (CPL)	*Carica papaya*	*sn*-1,3 specific	Papaya latex	(*53*)
Heterologous lipase	*Rhizopus oryzae* (rROL)	*sn*-1,3 specific	Amberlite^TM^ IRA 96	(*53*)

Genetic engineering techniques (*i.e*. rational design, directed evolution, *etc.*) have been developed to modify native lipases from different microorganisms. These have improved lipase activity, stability, regioselectivity and enantioselectivity (*25*). Additionally, genetically engineered lipases are also expected to reduce the cost of lipases and facilitate economically affordable enzymatic reactions (*2*). Other than lipases, genetic engineering techniques have also been conducted in lipid-containing plants to produce the desired composition of lipids or oils. Sunflower oil normally contains only 16 to 20% of oleic acid (*2*), but through mutagenesis, sunflower cultivars can contain 90% of oleic acid and less than 7% saturated fatty acids.

## SOURCES AND TYPES OF SUBSTRATES FOR MLM LIPid SYNTHESIS

Vegetable oil, fish oil, animal fats, single TAG molecules, or free fatty acids can be used as sources of LCFAs and MCFAs for MLM structured lipid synthesis. Oil that contains high concentrations of MUFAs or PUFAs at *sn*-2 position can be considered as LCFA source, and as a potential substrate. A higher concentration of oleic acid (C18:1) at *sn*-2 position can be found in several types of vegetable oil, including palm olein, olive oil, and canola oil (*54*, *55*). Linoleic acid (C18:2) can be found in soybean oil, cottonseed oil, sunflower oil and safflower oil, while fish oil products are widely known as sources of eicosapentaenoic (EPA, C20:5) and docosahexaenoic acid (DHA, C22:6). Moreover, palm kernel oil and coconut oil are reported as sources of MCFAs, containing higher amounts of lauric acid predominantly at *sn*-1,3 positions (*40*). Other potential substrates in MLM synthesis can be seen in Table 2 (*39*, *43*, *56*-*59*).

**Table 2:** Potential substrates for medium-long-medium (MLM) structured lipid synthesis

Fatty acid	Source	*w*(acid)/%	Reference
Oleic acid	Palm olein	39.8–46.0	(*56*)
	Canola oil	64.1	(*57*)
	Peanut oil	46.5	(*57*)
	Olive oil	55–83	(*57*)
	Rice bran oil	38–48	(*56*)
	Sesame seed oil	36.9–47.9	(*56*)
	Avocado oil	65.42	(*39*)
Linoleic acid	Soybean oil	48–59	(*56*)
	Cottonseed oil	46.7–58.2	(*56*)
	Sunflower oil	48.3–74	(*56*)
	Safflower seed oil	67.8–83.2	(*56*)
Linolenic acid	Flaxseed oil	50.28	(*43*)
EPA	Krill oil	14.3–28.0	(*58*)
	Menhaden oil	12.5–19.0	(*58*)
DHA	Tuna oil	21–42.5	(*58*)
	Anchovy oil	4.0–26.5	(*58*)
	Salmon oil	6.0–14.0	(*58*)
	Cod liver oil	6.0–18.0	(*58*)
Lauric acid	Palm kernel oil	45–55	(*56*)
	Coconut oil	45.1–53.2	(*56*)
	Babassu oil	40.5–55	(*56*)

Generally, vegetable oil or fish oil are sources of LCFAs for MLM structured lipid synthesis (Table 2) while vegetable oil, single TAG molecule (tricaprylin, tricaprin, *etc.*) and free fatty acids are MCFA sources. Several combinations of substrates for MLM lipid synthesis have been reported such as tricaprylin and trilinolenin (*59*), triolein and caprylic acid (*60*), olive oil and caprylic acid (*61*, *62*), modified pine nut oil and capric acid (*63*), canola oil and caprylic acid (*64*, *65*), soybean oil and caprylic acid (*7*, *66*-*68*), and corn oil and caprylic acid (*69*).

Besides the substrate availability and price, its purity is another factor that requires consideration. It has been reported that minor compounds found together with the lipids, such as hydroperoxides, phospholipids, emulsifiers, chlorophyll, carotenoids, lipid polymers, heavy metals, and even some antioxidants, have remarkable effect on the stability of lipases (*35*). Therefore, it is worth noting that the use of a high-quality initial substrate for MLM structured lipid synthesis can minimize enzyme stability loss.

The specificity of enzymes during MLM lipid synthesis may also be affected by the medium of reaction system. Generally, for lipase-catalyzed interesterification, MLM structured lipid synthesis can be performed in either solvent system (*i.e*. organic solvents) or solvent-free system. Although organic solvents are still permitted for ingredient preparation in food production, solvent-free system is more preferred in food industries to ease the separation processes, enhance the product quality and for environmental reasons (*26*, *70*). Nunes *et al.* (*17*) reported that a better incorporation of C8:0 and C10:0 into TAG was obtained in a solvent-free system than in the solvent system. However, the presence of organic solvent allows the reaction at higher temperatures (*26*). Higher temperatures reduce substrate viscosity, which entails reduced mass transfer and a higher reaction rate (*71*, *72*). On the other hand, higher temperatures also have a negative impact on enzyme inactivation. High temperature causes only partial unfolding of the enzyme structure, especially of the primary structure due to the damage of certain amino acids (*26*).

The use and selection of suitable organic solvent for MLM structured lipid synthesis must take into consideration the substrate and product solubility, hydrophobic behaviour of the solvent, reactivity, density, viscosity, surface tension, environmental effects, and cost (*26*). There is a report that organic solvents with log *P*>3 are the best solvents for the acidolysis of lard with capric acid (*73*). In another report, ionic liquid is used as alternative solvent in lipase-catalyzed reaction. The use of ionic liquid shows more technological advantages than organic solvents, such as selectivity enhancement, enzyme stability improvement, higher conversion rates, and better recyclability and recovery systems (*74*). However, there is still limited research using the ionic liquid in structured lipid production.

## METHODS OF ENZYME-CATALYZED MLM LIPID SYNTHESIS

The appropriate methods of MLM structured lipid synthesis will affect its purity (*75*). In general, enzyme-catalyzed MLM structured lipid synthesis can be obtained by direct or indirect interesterification. In direct interesterification, direct reaction between substrates and enzymes produces MLM structured lipids. Depending on the used substrate, direct interesterification includes acidolysis, transesterification and esterification. Alternatively, indirect interesterification or two-step reaction is a combination of two types of direct reactions to produce MLM structured lipids (*16*, *75*).

### Acidolysis

Acidolysis is a reaction between acylglycerol and free fatty acids. This reaction is mostly used for MLM structured lipid synthesis. Ifeduba and Akoh (*76*) used soybean oil rich in stearidonic and caprylic acids as a substrate with *Rhizomucor miehei* lipase as a catalyst. The result showed that structured lipids contained amount ratio of *r*=17–32.5% caprylic acid and 20.6–42.3% stearidonic acid. In another study, Caballero *et al.* (*39*) attempted to incorporate caprylic acid into avocado oil (2:1 substrate ratio) using Lipozyme TL IM and Lipozyme RM IM as catalysts. The reaction was conducted in a solvent-free system at 10–50 °C for 24 h. The highest amount ratio of caprylic acid at *sn*-1,3 positions obtained using Lipozyme TL IM as a catalyst was 29.2%.

Repeated reactions can be used to increase the yield of MLM structured lipid synthesis. The repeated reactions effectively enhance the incorporation of caprylic acid at *sn*-1,3 positions. Lai *et al.* (*77*) reported that 30.5% of caprylic acid was incorporated into refined bleached deodorized palm olein. However, acyl migration that occurs in acidolysis can result in reduced purity of MLM structured lipid yield. Acyl migration is generally defined as the migration of the acyl group from *sn*-2 to *sn*-1,3 position (*78*). This phenomenon has been reported to be influenced by the temperature, types of immobilization carrier and organic solvent (*79*).

### Transesterification

Transesterification is the reaction of exchange of two acyl groups between two ester molecules or TAGs. Depending on the substrates used in the reaction, there are three types of transesterification: (*i*) reaction between two types of vegetable oil, (*ii*) reaction between vegetable/fish oil and single TAG molecule, and (*iii*) reaction between two single TAG molecules. For the MLM structured lipid synthesis using the first type of reaction, Zhao *et al.* (*80*) used *Cinnamomum camphora* seed oil and camellia oil catalyzed by Lipozyme RM IM. The produced structured lipid had the oleic acid predominantly at *sn*-2 position (88.69%) and MCFA at *sn*-1,3 position (68.05%). A reaction between linseed oil and tricaprylin catalyzed by Lipozyme TL IM for MLM synthesis is an example of the second type of transesterification (*81*). Here, the amount ratio of caprylic acid-linolenic acid-caprylic acid was the highest (35.34–35.45%). Caprylic acid-linoleic acid-caprylic acid, and caprylic acid-oleic acid-caprylic acid were also produced at amount ratios of 4.09–4.19 and 8.44–8.53%, respectively. As an example of esterification type three, Bai *et al.* (*59*) reported the utilization of tricaprylin and trilinolenin as substrates, and either Lipozyme RM IM or Novozyme 435 as a catalyst. Caprylic acid-linolenic acid-caprylic acid and caprylic acid-linolenic acid-linolenic acid were reported to be the dominant products. It is conclusively considered that the MLM structured lipid synthesis using transesterification may produce various TAG species as compared to other methods. Therefore, purification of structured lipids is necessary to eliminate byproducts such as free fatty acids, monoacylglycerols (MAG) and diacylglycerols (DAG), and to obtain a higher MLM structured lipid yield. Purification methods of structured lipids can be short-path distillation (*82*), membrane technology (*83*), and solvent extraction (*50*, *84*, *85*). In solvent extraction, hexane is used under alkaline condition to purify the structured lipid. Lu *et al.* (*50*) conducted a two-step purification using deacidification and silica gel absorption. After the purification, the product had reduced acid and peroxide values, and DAG content.

### Esterification

Glycerol and free fatty acids are generally used as substrates in esterification reaction. This reaction produces a highly pure MLM with fewer byproducts than acidolysis. The esterification products can be at a higher risk of degradation as they do not contain any natural antioxidant from vegetable oils (*16*). Therefore, in the post-reaction step, an antioxidant has to be added to maintain the product stability. Arifin *et al.* (*86*) used glycerol and a mixture of stearic and capric acids as substrates and Lipozyme RM IM as a catalyst. The result showed that 58% medium and long-chain triacylglycerol (MLCT) was produced under optimum conditions (13.6–14.0 h reaction time, 7.9–8.0% (*m*/*m*) enzyme load, and 3:1 fatty acids/glycerol amount ratio). A similar study was also conducted using caprylic, capric and oleic acids, and glycerol (*87*). The use of non-specific lipase (Novozyme 435) in this reaction produced 72.19% MLCT (*87*). It has been reported that non-specific lipase is more suitable than specific *sn*-1,3 lipase for esterification during MLM structured lipid synthesis (*87*). This is due to acyl migration that can occur in specific *sn*-1,3 lipase-based MLM structured lipid synthesis.

### Two-step reaction

Two-step reaction is mostly applied when using PUFA as substrates. The hydrolysis of PUFA by fungal lipase is more difficult than other fatty acids. However, this method has been reported to produce highly pure MLM structured lipids (*16*, *75*). There are various strategies for structured lipid synthesis based on two-step reaction. The first approach is a combination of esterification and acidolysis (*16*). In the initial step, the esterification occurs between glycerol and PUFA producing tri-PUFA. Then, acidolysis between tri-PUFA and MCFA yields MLM TAG. Kawashima *et al.* (*88*) produced MLM structured lipids using this method and EPA, gamma-linolenic acid (GLA), arachidonic acid (AA), DHA and caprylic acid. Esterification was catalyzed by Novozyme 435 to produce tri-GLA, tri-AA, tri-EPA and tri-DHA. Furthermore, acidolysis between caprylic acid and tri-GLA/tri-AA/tri-EPA or tri-DHA was conducted to produce MLM structured lipids with mass fractions of 58, 87, 86 and 19%, respectively (*88*).

Meanwhile, in the second approach a combination of alcoholysis and acidolysis was used (*31*, *89*). Firstly, vegetable or fish oil with high concentration of PUFA at *sn*-2 position reacted with an alcohol to produce 2-monoacylglycerol (2-MAG). Then, 2-MAG reacted with MCFA to produce MLM structured lipids. This strategy was adopted to produce caprylic acid-gamma linoleic acid-caprylic acid from borage oil (*88*). Muñío *et al*. (*51*) used similar method to produce caprylic acid-PUFA-caprylic acid from fish oil (cod liver and tuna oil). In alcoholysis, various *sn*-1,3-specific lipases were employed to produce 2-MAG. The highest concentration of 2-MAG was obtained using lipase D (*Rhizopus oryzae* lipase). However, Novozyme 435 showed better operational stability than lipase D. 2-MAG was separated using a solvent extraction with ethanol and hexane. Furthermore, 2-MAG reacted with caprylic acid to produce MLM TAG. The product contained 45% PUFA at *sn*-2 position. The incorporation of caprylic acid was 64%, and about 98% of this incorporation were found at *sn*-1,3 position. A similar study was also performed using cod liver oil and caprylic acid (*90*) and about 38% DHA were found at *sn*-2 position and 60% caprylic acid at *sn*-1,3 position.

Morales-Medina *et al.* (*91*) conducted an alternative strategy to two-step reaction. In the first step, an esterification reaction catalyzed by Novozyme 435 was performed using glycerine and caprylic acid to produce 1,3-dicaprylin, which reacted with LCFA to produce MLM TAG. This method increased regiodistribution of fatty acids by 72% as compared to direct reactions (*91*).

## REACTOR FOR MLM STRUCTURED LIPID SYNTHESIS

In lipid modification process, the selection of the reactor is of importance to produce the desired product in high quantities. Several factors important for the selection of reactor are: flexibility, efficiency, quality of the final product, stability and reusability of enzymes, and cost (*92*). In MLM lipid synthesis, there are two types of reactor systems commonly used: batch and continuous system (*93*, *94*).

The batch reactor system is widely used to collect preliminary data on bench-to-pilot plant scale. Sometimes, this reactor is also used for production of TAG in small quantities, especially for characterization of newly isolated enzymes for process development of new products. This reactor requires simple equipment and operation (*94*). Limitations of this system are the difficulty to control heat transfer, and variations in batch-to-batch operation. On industrial scale, such repetitive actions in start and end procedure in every batch will result in the accumulation of unproductive time and increased labour cost. Substrate ratio, enzyme load, stirring rate, temperature, and reaction time influence the yield of MLM lipid synthesis in batch reactor (*68*). Wang *et al.* (*65*) reported that incorporation of caprylic acid in a batch production was influenced by substrate amount ratio. The maximum incorporation of caprylic acid was 45.31% at amount ratio of caprylic acid and canola oil 4:1. A higher amount ratio than this value was not found to increase the incorporation of caprylic acid. Additionally, the increase of caprylic acid amount ratio in the reaction system might induce enzyme inactivation (*68*). Wang *et al.* (*65*) also reported that loading of Lipozyme RM IM higher than 10% decreased the incorporation of caprylic acid, which is in agreement with another study where maximum incorporation of caprylic acid in acidolysis was 10% of lipase load (*95*). On the other hand, a higher reaction temperature caused an increasing rate of reaction, but also showed enzyme denaturation and acyl migration (*96*). In general, a higher possibility of acyl migration also occurred at longer batch reaction time (*97*). Moreover, higher stirring rate caused destruction of immobilized enzymes, while lower stirring rate affects substrate homogeneity (*71*). Table 3 (*15*, *17*, *40*, *44*, *48*, *49*, *64*, *80*, *98*-*101*) shows relevant studies about batch production of structured lipids.

**Table 3:** Conditions in batch reactor for medium-long-medium (MLM) lipid synthesis

Type of reaction	Substrate	*r*(substrate)	Immobilized enzyme	Enzyme loading/%	Temperature/°C	Stirring rate/rpm	Reaction time/h	Yield	Reference
Acidolysis	Olive oil+caprylic acid	1:2	heterologous *Rhizopus oryzae* lipase	5	40	400	24	*r*(caprylic acid)=21.6%	(*17*)
Acidolysis	Virgin olive oil+caprylic acid or capric acid	1:2	Lipase Lip2	5	40	–	48	*r*(caprylic and capric acid)=25.6 and 21.3%	(*44*)
Acidolysis	Mustard oil+capric acid	1:3.5	Lipozyme TL IM	8.8	39.5	570.8	21.1	*w*_max_(capric acid)=23%	(*48*)
Acidolysis	Menheden oil+capric acid or ethyl caprate	1:3	Lipozyme 435	10	60	250	16	*r*(ethyl caprate and capric acid)=30.76 and 19.50%	(*49*)
Acidolysis	Palm olein+caprilyc acid and capric acid	4:3:3	Lipozyme RM IM	10	90	–	24	*r*(caprylic and capric acid)=36 and 40%	(*40*)
Acidolysis	Canola oil+aprylic acid	1:3	Lipozyme TL IM	12	55	200	15	*r*(caprylic acid)=37.2%, *w*(MLM lipid)=21.2%	(*64*)
Acidolysis	*Echium* oil+lauric acid	1:5	Lipozyme RM IM	10	50	200	4	*r*(lauric acid)=42.8%	(*98*)
Acidolysis	Microbial oil from *Mortierella alpina*+caprylic acid	1:3	Lipozyme RM IM	6	60	300	6	*r*(caprylic acid)=20.14%, *w*(MLM (CAC) lipid)=11.23%	(*99*)
Transesterification	RBD palm oil+RBD palm kernel oil	10:90	Lipozyme TL IM	5	50	350	7.26	*w*(MLCT including MLM lipid)=60%	(*100*)
Transesterification	*Cinnamomum camphora* seed oil+camellia oil	1:1.5	Lipozyme RM IM	10	60	200	3	*w*(MLCT)=55.81% with predominantly oleic acid (*w*=88.69%) at *sn-*2 position, and *w*(MCFA)=68.05% at *sn*-1,3 position	(*80*)
Acidolysis and transesterification	Evening primrose oil (EPO)+tricaprylin or caprylic acid	Acidolysis (EPO:caprylic acid=1:3) Transesterification (EPO:tricaprylin=1:2)	*Rhizopus delemar* lipase (RDL), *Rhizomucor miehei* lipase (RML)	10	40	300	24	*w*(MLM lipid by transesterification)=23% (RDL) and 28% (RML), *w*(MLM lipid by acidolysis)=32–38% (RDL) and 25–28% (RML)	(*15*)
Two-step: 1) alcoholysis 2) esterification	1) Microalgae oil+ethanol 2) 2-MAG (from alcoholysis)+ caprylic acid	1) 1:24 2) 1:3	Lipozyme TL IM	10	1) 25 2) 40	300	1) 12 2) 24	*w*(MLM lipid with mainly caprylic-LCFA-caprylic)=43.01%, *w*(MLM lipid with mainly caprylic-SDA-caprylic)=23.88%	(*101*)

RBD=refined, bleached and deodorized, CAC=caprylic-arachidonic-caprylic, MLCT=medium-long chain triacylglycerol, MCFA=medium chain fatty acid, LCFA=long chain fatty acid, SDA=stearidonic acid

In continuous reactor system, substrate is fed continuously into the reactor. Moreover, the remaining or unreacted substrate together with the product flows continuously out of the reactor. Hereby, residence or retention time plays an important role for obtaining a higher reaction rate. In general, there is a distinction between a continuous stirred tank reactor (CSTR) and plug flow reactor or packed bed reactor (PBR). In CSTR, the samples are homogeneously mixed, and the temperature is maintained at each point inside the reactor. PBR is a reactor system where the substrate mixture passes through a reactor tube containing immobilized enzyme molecules. PBR has several advantages such as ease of operation, better product control, and high reaction rate and mass transfer (*93*). This reactor is commonly used for MLM structured lipid synthesis in continuous system (*102*-*104*).

Substrate flow in PBR system can be performed either downward or upward through the application of metering pump. For downward flow, the substrate enters from the top of the reactor and the product will exit from the bottom of the reactor collected in the product reservoir. This method causes faster flow rates (thus shorter residence time) due to the gravity effect besides pressure gradient produced by the work of the pump. Contrary to the downward flow, in the upward flow the substrate flows from the bottom of the reactor and the product will exit through the top of the reactor. This method is more common in industrial applications (*105*).

In addition to the two aforementioned flow strategies in PBR, a substrate flow can also be maintained using recycling method (*93*, *106*). The recycling method is carried out by streaming the mixture of unreacted substrate and product out of the reactor and pumping it back into the reactor. Generally, similar to batch reactor, this method uses stirrers for both reactor and substrate reservoir to facilitate continuous stirring and maintaining substrate homogeneity (*107*). The advantage of this strategy is the increase of product yield as substrate is repetitively exposed to and catalyzed by the enzyme. Here, the product will be taken periodically from the substrate reservoir.

**Fig. 2:**
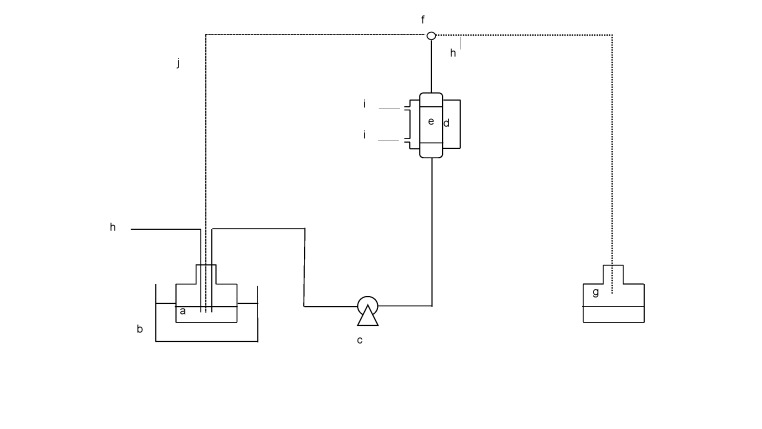
Packed bed reactor with single (continuous) and recycling (discontinuous) flow: a) substrate reservoir, b) reactor temperature control, c) peristaltic pump, d) water jacket, e) packed bed reactor, f) three-way valve, g) product reservoir, h) sampling, i) cooling/heating water, and j) recirculation (*103*)

Paez *et al.* (*103*) reported the MLM structured lipid synthesis using caprylic acid and cod liver oil as substrates and Lipozyme IM as a catalyst. The reaction was performed in three reactor configurations: batch, PBR with recycling flow (discontinuous) flow, and PBR with continuous flow (Fig. 2). In Fig. 2, the dashed line shows the substrate flow in recycling mode (discontinuous), whereas the dotted line shows the substrate flow in continuous mode. A similar study was also conducted using tuna oil and caprylic acid to synthesize MLM TAG in a packed bed reactor (*52*). The result showed that incorporation of higher amount of caprylic acid into tuna oil was obtained in discontinuous flow than in continuous flow. In contrast, Gonzáles Moreno *et al*. (*108*) reported that incorporation of caprylic acid into fish oil using PBR was higher in continuous flow than in the discontinuous flow (recycling flow). In addition, a higher EPA content was achieved in continuous flow. It seems that other factors such as enzyme and substrate types, and substrate amount ratio might also play an important role in MLM lipid synthesis. Table 4 (*42*, *52*, *63*, *109*, *110*) summarizes literature about MLM structured lipid synthesis in a continuous system.

**Table 4:** Conditions in a continuous reactor for medium-long-medium (MLM) type structured lipid synthesis

Type of reaction	Packed bed reactor	Substrate	Substrate ratio	Enzyme	Enzyme loading/g	Temp./ °C	Flow rate/ (mL/min)	Residence time/min	Yield	Reference
Acidolysis	id=0.48 cm; *l*=7.62 cm	Modified pine nut oil+capric acid	1:5 (*n*_A_/*n*_B_)	Lipozyme RM IM	0.7	60	0.6	–	MLM lipids were dominated by TAG species with equivalent carbon number (ECN)=38	(*63*)
Transesterification	id=1.15 cm; *l*=20 cm	Soybean oil+ MCT (*x*=60% caprylic and 40% capric acid)	1:1 (*m*_A_/*m*_B_)	Lipozyme TL IM		55	0.426	30–40	The interesterfication degree of reaction was more than 50%	(*42*)
Transesterification	id=26 mm; *l*=40 cm	Fish oil+MCT (*x*=60% caprylic and 40% capric acid)	1:1 (*n*_A_/*n*_B_)	Lipozyme TL IM		60	–	30–40	The interesterfication degree of reaction was more than 80%	(*109*)
Acidolysis	id=1.8 cm; *l*=18 cm	Borage oil+caprylic acid	1:2 (*n*_A_/*n*_B_)	*Candida rugosa* lipase (Lipase-OF) and *Rhizopus oryzae* lipase (Ta-lipase)	15	30	0.075	–	Produced *r*=44.5% caprylic-ɣ linolenic-caprylic (CGC)	(*110*)
Acidolysis	id=2.5 cm; *l*=25 cm	Tuna oil+caprylic acid	1:6 (*n*_A_/*n*_B_)	Lipase Rd, lipase Palatase	14.1	30	0.417–1	–	Produced MLM lipids with 50% of caprylic acid and 16–20% of DHA	(*52*)

MCT=medium chain triacylglycerol

## APPLICATIONS OF MLM STRUCTURED LIPIDS

The digestion of lipids in human body occurs when the lipase is present. The lipases involved in this process are lingual, gastric, pancreatic and co-pancreatic, found in the mouth, stomach, and small intestine, respectively (*55*). However, the major digestion and absorption of lipid derivatives are located at the small intestine, especially duodenum, in the presence of pancreatic and co-pancreatic lipase (*55*). Pancreatic and co--pancreatic lipases specifically hydrolyze fatty acids at *sn*-1 and *sn*-3 positions, respectively (*55*). MLM TAG are hydrolyzed by pancreatic lipase to produce 2-MAG and two free fatty acids. The rate of hydrolysis of the MLM (M=C8:0 or C10:0, L=C18:2) is twofold higher than that of LML lipids (long-medium-long) (*111*). In animal studies, Ikeda *et al.* (*112*) showed that the absorption of linoleic acid was higher than of trilinolein in MLM (capric acid-linoleic acid-capric acid) lipids. This was probably due to faster hydrolysis rate of MCFA. In addition, the absorption of LCFA is higher when they are located at the *sn*-2 position (*113*, *114*). Saturated LCFA, such as palmitic and stearic acids, absorb better the total MLM lipids than LML lipids (*115*). In LML lipids, saturated fatty acids escape from the outer position of TAG (*sn*-1 and *sn*-3 positions) during lipolysis, and form calcium soaps that are excreted through the faeces (*5*). In addition, human body absorbes more easily the PUFA at the *sn*-2 position than 2-MAG. Therefore, it is important to maintain LCFA at the *sn-*2 position.

Various studies have explored the potential applications of MLM lipids in food products. Currently, there is no commercial pure MLM lipid product on the market. Small quantities of MLM lipids can be found commercially in Resetta, a commercial MLCT product manufactured by Nisshin Oillio Group Ltd. Tokyo, Japan. This product was categorized as Food for Specific Health Uses (FOSHU) in Japan in 2002, and received Generally Recognized as Safe (GRAS) status from Food and Drug Administration (FDA) in 2006 (*16*). This product is composed of soybean oil or cottonseed oil as sources of LCFA and palm kernel oil or coconut oil as sources of MCFA. It consists of LLL (49.5–52.7%), LLM or LML (37.3–39.63%), LMM or MLM (8.6–9.34%), and MMM (0.1–0.2%) lipids. This product is also known as a healthy cooking oil which is stable at 200 °C for 30 min. In another report, Jennings *et al.* (*116*) showed that MLM structured lipids (caprylic acid-oleic acid-caprylic acid) can be used as cooking oil for sweet potato chips at 165–185 °C for 20–60 s. Sensory evaluation using triangle test showed no significant difference between sweet potato chips fried using MLM structured lipids and rice bran oil (*116*).

MLM TAG can also be used as an ingredient for other food products such as energy bars, mayonnaise, margarine and beverages. MLM structured lipids from rice bran oil and rice bran oil were reported to be used in energy bars (*116*). Jacobsen *et al.* (*117*) reported that mayonnaise from structured lipids has a lower oxidative stability than mayonnaise from traditional sunflower oil or from chemically randomized lipids due to a lower tocopherol content, a higher initial level of lipid hydroperoxides, and a higher proportion of secondary volatile oxidation compounds. Regarding rheological properties, the type of lipid used did not affect the gel strength or the phase angle of mayonnaise (*117*). Osborn *et al.* (*118*) used structured lipids from canola oil and caprylic acid for the formulation of chocolate--flavoured nutritional beverages. The results showed that substituting the unmodified canola oil with structured lipids significantly improved the perception of sweetness, and decreased bubble formation. In addition, the use of MLM lipids does not change all attributes of the beverage formulation. Milk drinks from structured lipids show similar viscosity to the milk drink from sunflower oil, while lower than milk drink from randomized lipids (*119*). Moore and Akoh (*120*) used MLM product from coconut oil and sunflower oil with high oleic acid content to formulate edible film for sport nutrition products. In nutraceutical products, MLM lipids enhance the lymphatic transport and the portal absorption of the poorly water-soluble drugs and halofantrine in animal study compared to sunflower oil (*12*, *121*).

## CONCLUSIONS

Medium-long-medium (MLM) structured lipids have a great potential as functional ingredients in food and nutraceutical products. A careful selection of appropriate substrate, enzyme, reactor configuration and reaction conditions is necessary to increase the efficiency of MLM lipid synthesis, especially to increase yield and reduce production cost. Generally, the optimal reaction conditions for MLM lipid synthesis in batch reactor system are reaction temperature 40–60 °C, enzyme load 10% (*m*/*m* of total substrates), stirring rate 200–400 rpm and reaction time 4–24 h. Immobilized lipases are widely used as catalysts for structured lipid synthesis. The reaction conditions varied depending on the reactor configuration, especially for continuous reaction. The exploration of other potential sources of substrates (lipids) and enzymes is required to increase MLM lipid yield and reduce cost. It is of importance to make the application of MLM lipids feasible for food industries.
